# BEREN: a bioinformatic tool for recovering giant viruses, polinton-like viruses, and virophages in metagenomic data

**DOI:** 10.1093/bioadv/vbaf284

**Published:** 2025-11-08

**Authors:** Benjamin Minch, Mohammad Moniruzzaman

**Affiliations:** Department of Marine Biology and Ecology, Rosenstiel School of Marine, Atmospheric, and Earth Science, University of Miami, Miami, FL 33149, United States; Department of Marine Biology and Ecology, Rosenstiel School of Marine, Atmospheric, and Earth Science, University of Miami, Miami, FL 33149, United States

## Abstract

**Motivation:**

Viruses in the kingdom Bamfordvirae, specifically giant viruses (NCLDVs) in the phylum Nucleocytoviricota and smaller members in the Preplasmiviricota phylum, are widespread and important groups of viruses that infect eukaryotes. While viruses in this kingdom, such as giant viruses, polinton-like viruses, and virophages, have gained large interest from researchers in recent years, there is still a lack of streamlined tools for the recovery of their genomes from metagenomic datasets.

**Results:**

Here, we present, BEREN, a comprehensive bioinformatic tool to unlock the diversity of these viruses in metagenomes through five modules for NCLDV genome, contig, and marker gene recovery, metabolic protein annotation, and Preplasmiviricota genome identification and annotation. BEREN’s performance was benchmarked against other mainstream virus recovery tools using a mock metagenome, demonstrating superior recovery rates of NCLDV contigs and Preplasmiviricota genomes. Overall, BEREN offers a user-friendly, transparent bioinformatic solution for studying the ecological and functional roles of these eukaryotic viruses, facilitating broader access to their metagenomic analysis.

**Availability and implementation:**

BEREN is available at https://gitlab.com/benminch1/BEREN, and results from testing BEREN on a real-world metagenome are available in the Supplementary Files.

## 1 Introduction

The Eukaryotic viruses are widespread and important members of the global virosphere, infecting both charismatic macrofauna such as pigs and smaller microeukaryotes like photosynthetic algae. One of the major kingdoms of these viruses is Bamfordvirae, which includes the Nucleocytoviricota phylum (giant viruses) (Aylward *et al.* 2021) and the closely related Preplasmiviricota phylum, which contains virophages, polintons, and polinton-like viruses (Koonin *et al.* 2024). Recent research has highlighted the widespread presence of viruses from these two phyla of double-stranded DNA viruses in diverse ecosystems, including both terrestrial and aquatic environments (Bellas and Sommaruga 2021; Minch *et al.* 2023; Schulz *et al.* 2018, 2020). These viruses also often infect environmentally important microeukaryotic hosts such as the bloom-forming algae Emiliania huxleyi and Phaeocystis globosa (Bratbak *et al.* 1993; Juneau *et al.* 2003; Vincent *et al.* 2021).

While many bioinformatic tools exist for recovering viruses from metagenomes, most of these tools have a strong emphasis on prokaryotic viruses such as phages infecting bacteria (Wu *et al.* 2024). Some recent tools, such as geNomad and IPEV (Camargo *et al.* 2024; Yin *et al.* 2024), can identify dsDNA eukaryotic viruses, but these tools often lack specificity as to the identity of the eukaryotic virus or the ability to recover full genomes of these viruses for comprehensive downstream analysis. The recovery of full genomes is especially of concern for giant viruses, as their genomes are often too large to be assembled into a single contig through short-read sequencing, and no current tool is fit to recover full genomes. In addition, these tools do not take into account the ever-expanding diversity of these viruses, such as the newly discovered Mirusviricota phylum (Gaïa *et al.* 2023), Egovirales order (Gaïa *et al.*, 2024), or Mriyaviricetes class (Yutin *et al.* 2024).

There is currently no consensus methodology or comprehensive tool for recovery of these viruses (Bellas and Sommaruga 2021; Moniruzzaman *et al.* 2020; Pitot *et al.* 2024; Schulz *et al.* 2020), and many of the proposed methods require extensive bioinformatic expertise and manual screening. Here we present BEREN (Bioinformatic tool for Eukaryotic virus Recovery from Environmental metagenomes), a “one-stop-shop” for uncovering the diversity and metabolic potential of giant viruses, polinton-like viruses, and virophages in any metagenomic sample. This tool both opens up the realm of Bamfordvirae viruses to interested researchers and provides a streamlined methodology to increase repeatability. We demonstrate that this tool outperforms available virus recovery tools in recovering NCLDV contigs and Preplasmiviricota genomes, as well as provides extra functionality integrated in the pipeline, such as genome binning, taxonomy, decontamination, and metabolic annotation.

## 2 Methods

### 2.1 The BEREN tool

**Figure 1.**
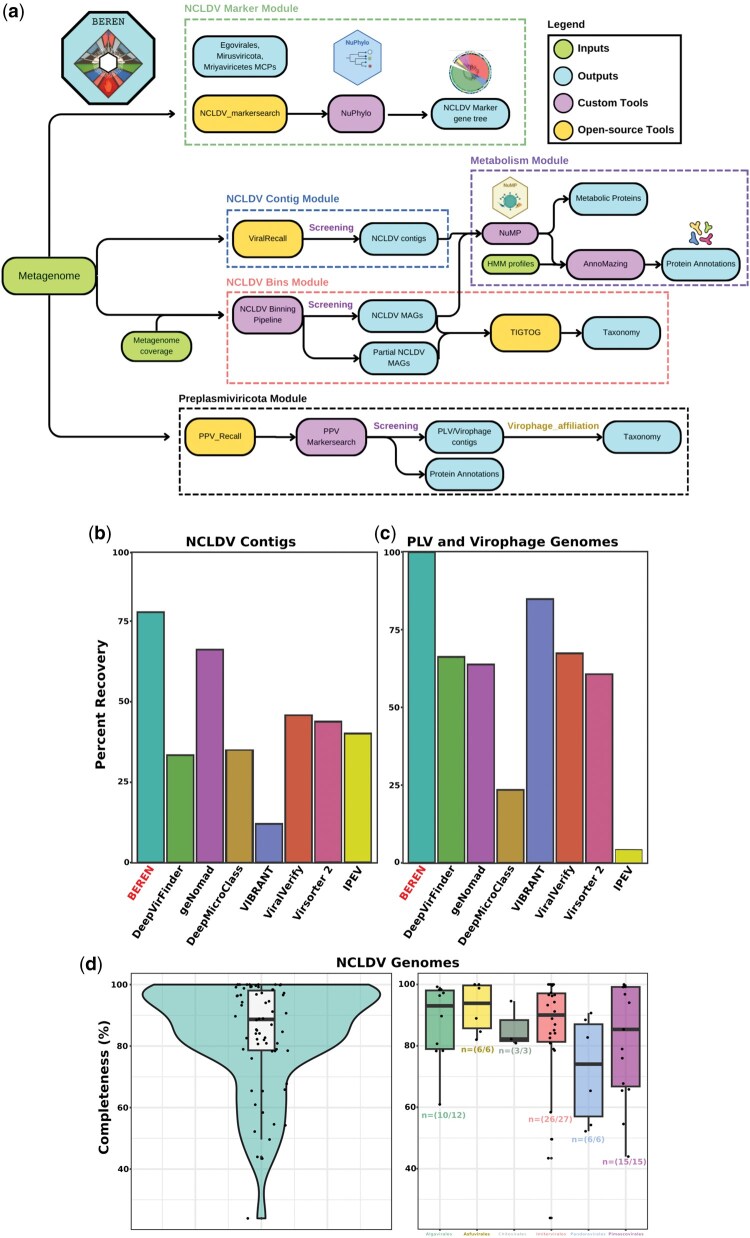
The BEREN pipeline and benchmarking. (a) BEREN consists of 5 major modules, each comprising multiple open-source tools as well as custom scripts. The input of a metagenome can yield NCLDV markers, contigs, and genomic bins, Preplasmiviricota genomes, as well as the metabolic potential and protein annotation of these viruses. The recovery of (b) NCLDV contigs and (c) PLV and virophage genomes was benchmarked against popular virus identification tools. Each tool was run with default settings, and total recovery is shown here. (d) The completeness of the recovered NCLDV genomes from BEREN was tested by comparing recovered genomes to those used to build the mock metagenome. Completeness is defined as the percentage of the total length of the genome recovered. This information is also provided for each NCLDV order, with the number of recovered genomes in that order below the boxplots.

The BEREN tool comprises five different modules that can be run independently or all together on a metagenomic dataset. These modules include (1) NCLDV markers, (2) NCLDV contigs, (3) NCLDV bins, (4) metabolism and protein annotation, and (5) Preplasmiviricota identification. The tool is publicly available at: https://gitlab.com/benminch1/BEREN. Detailed instructions and necessary scripts for installation and database downloads are also provided there, along with default parameters for the tools integrated in the BEREN workflow (Fig. 1).

#### 2.1.1 NCLDV marker module

The main purpose of the NCLDV marker module is to gain insight into the full diversity of NCLDVs within a given metagenomic dataset, including those that remain elusive to genome binning methods due to low coverage or abundance, as well as newly discovered NCLDV relatives such as Egoviruses, Mirusviruses, and Mriyaviruses. The NCLDV marker module first predicts proteins from the metagenomic assembly using prodigal-gv (Camargo *et al.* 2024; Hyatt *et al.* 2010). It then searches for NCLDV marker genes with the NCLDV_markersearch script (Aylward *et al.* 2021). The marker genes searched for include the NCLDV major capsid protein (MCP), DEAD/SNF2-like helicase (SFII), DNA-directed RNA polymerase beta and alpha subunits (RNAPS and RNAPL), DNA polymerase family B (PolB), Transcription initiation factor IIB (TFIIB), DNA topoisomerase II (TopoII), Packaging ATPase (A32), and the Poxvirus Late Transcription Factor VLTF3 (VLTF3). These markers were chosen based on their near universality in NCLDV genomes and their use in phylogenetic placement of these viruses (Aylward *et al.* 2021).

After storing all markers in separate protein FASTA files, this module will use the newly discovered PolB markers in the target metagenome dataset to build a phylogenetic tree (see Supplementary Methods). The sequences are first aligned using MAFFT (Katoh *et al.* 2002) to a custom set of reference NCLDV PolBs representing all NCLDV families, as well as a set of PolBs from bacteria and eukaryotes for quality filtering. The alignment is then trimmed with trimAL using the “-gt 0.1” parameter (Capella-Gutiérrez *et al.* 2009), and a phylogenetic tree is constructed using fasttree (Price *et al.* 2010).

#### 2.1.2 NCLDV contig module

While NCLDV genome recovery is an obvious goal of many researchers wanting to study these viruses, recovery of full genomes might not always be possible due to fragmented assemblies and known chimerism of NCLDVs (Moreira and Brochier-Armanet 2008; Yu *et al.* 2024). In the NCLDV contig pipeline, NCLDV contigs are retrieved using ViralRecall (Aylward and Moniruzzaman 2021), which identifies NCLDV contigs based on homology to known NCLDV proteins. The identified contigs are screened for an NCLDV score chosen by the user (default of 3) and a length of >10kbp. The method for deriving these scores is described in Aylward and Moniruzzaman (2021).

#### 2.1.3 NCLDV bins module

This module is designed to recover both high-quality and partial NCLDV genomes through a genome binning, screening, and cleaning pipeline. First, metagenomic contigs are binned using metabat2 (Kang *et al.* 2019), and then potential NCLDV bins are identified using the NCLDV markersearch script (Aylward *et al.* 2021) after proteins are predicted using prodigal-gv (Camargo *et al.* 2024). Bins with ≥1 hit to an NCLDV marker gene are retained for further analysis. These bins are then screened using ViralRecall in “contig” mode (Aylward and Moniruzzaman 2021) to confirm they are NCLDVs. Bins with negative ViralRecall scores (those with more hits to cellular protein groups than viral ones) are further investigated to assess whether they represent partial NCLDV genomes.

After this screening, another screening is performed to further separate “high-quality” and “partial” NCLDV bins. High-quality NCLDV bins are defined as having at least 3 of the following marker genes (PolB, MCP, A32, VLTF3, SFII) so they can be easily used in a concatenated phylogeny (Aylward *et al.* 2021). These markers were chosen due to their highly conserved nature in NCLDVs (Aylward *et al.* 2021; Moniruzzaman *et al.* 2020). Both partial and high-quality bins are then processed using ViralRecall in “contig” mode, which screens individual contigs within the bins to remove potential cellular contamination. Taxonomy for these bins is assigned using TIGTOG, a tool that predicts taxonomy through a machine learning approach (Ha and Aylward 2024).

The NCLDV bins module can also potentially recover Mirusvirus and Mryiavirus genomes. The supplementary text discusses this in more detail (Fig. S1).

#### 2.1.4 Metabolism and protein annotation module

Many NCLDVs encode genes for a wide range of cellular metabolic processes, potentially enabling them to reprogram cellular metabolism in myriad different ways, which is unprecedented in the virus world (Minch and Moniruzzaman 2025; Moniruzzaman *et al.* 2023). The metabolism and protein annotation module attempts to uncover these unique functions of NCLDVs through HMM-based protein annotation using multiple databases. Using recovered NCLDV contigs, NCLDV high-quality bins, and partial bins, this module first annotates proteins using Pfam (Mistry *et al.* 2021), GVOG (Aylward *et al.* 2021), and KEGG (Kanehisa *et al.* 2017) databases. Metabolic proteins of interest are parsed into a separate file using a custom script (github.com/BenMinch/nump). Resulting protein annotations can be used to analyze the functional profiles of the whole NCLDV community or individual NCLDV genomes.

#### 2.1.5 Preplasmiviricota module

Virophages and Polinton-like viruses (PLVs) are all part of Preplasmiviricota, a sister phylum to NCLDVs (Koonin *et al.* 2024). These smaller eukaryotic viruses are widespread in aquatic ecosystems (Bellas and Sommaruga 2021) and often interact with NCLDVs (Roux *et al.* 2017), making their recovery important for understanding eukaryotic virus ecology. The Preplasmiviricota module effectively recovers and identifies these two types of viruses (Virophages and PLVs) in the Preplasmiviricota phylum in metagenomic datasets (see supplemental methods).

First, contigs potentially belonging to viruses in this phylum are screened using a modified version of ViralRecall with new protein profiles common across diverse virophage and PLV members (Stephens *et al.* 2024). Contigs with a positive score are then screened to only keep contigs with a major capsid protein as well as one other marker gene (integrase, minor capsid protein, and Ftsk ATPase). Taxonomy is assigned to these contigs using the Virophage_affiliation script (Roux *et al.* 2017). Protein annotations for these contigs are performed using both the Pfam database (Mistry *et al.* 2021) and a custom HMM profile of environmental virophage and PLV protein clusters (Bellas and Sommaruga 2021).

### 2.2 Benchmarking BEREN

#### 2.2.1 Mock metagenome creation

To test the capabilities of the BEREN tool at recovering diverse NCLDV and Preplasmiviricota members, a mock metagenome was created using CAMISIM (Fritz *et al.* 2019) [size = 3, 150 bp mean length]. To create the mock community, 100 genomes of unique virophages and PLVs (Bellas and Sommaruga 2021) were obtained (dereplicated at 80% ANI using dRep (Olm *et al.* 2017)) as well as 69 unique NCLDV genomes from the GOEV database (Gaïa *et al.* 2023)(637 total contigs), representing 3 from each major NCLDV family, covering all NCLDV orders. Ten bacterial genomes were also used to simulate ubiquitous bacterial populations within environmental metagenomic samples, and these genomes were obtained from the GORG tropics database (Pachiadaki *et al.* 2019).

#### 2.2.2 Benchmarking BEREN and other virus recovery tools

The BEREN tool was benchmarked against a set of other popular virus recovery tools, including DeepVirFinder (Ren *et al.* 2020), geNomad (Camargo *et al.* 2024), DeepMicroClass (Hou *et al.* 2024), VIBRANT (Kieft *et al.* 2020), ViralVerify (Antipov *et al.* 2020), Virsorter2 (Guo *et al.* 2021), and IPEV (Yin *et al.* 2024). While most of these tools were built with prokaryotic viruses in mind, they can often perform moderately well at recovering eukaryotic viruses (Camargo *et al.* 2024).

All tools were run with default settings using the mock metagenome as input. Many of these tools (with the exception of geNomad) will not explicitly discriminate between NCLDV and Preplasmiviricota viruses, so sequences identified as viral were run through the BEREN pipeline to parse these two groups apart. For tools using deep learning (DeepVirFinder, DeepMicroClass, and IPEV), a score cutoff of 0.9 was used to determine viral sequences. The percent recovery of NCLDV contigs, as well as Preplasmiviricota genomes, was evaluated for each tool.

#### 2.2.3 Benchmarking BEREN for NCLDV genome completeness

To assess the ability of BEREN to recover high-quality “complete” NCLDV genomes, the NCLDV bin module was run on the mock metagenome. Each recovered genome bin was compared to the known genomes in the mock community using BLASTn (Camacho *et al.* 2009). Completeness was defined to be the percentage of the total genome length recovered by BEREN.

## 3 Results

### 3.1 BEREN exceeds other automated virus detection tools in NCLDV and preplasmiviricota recovery

A benchmarking of the BEREN tool against other popular viral recovery tools (DeepVirFinder, geNomad, DeepMicroClass, VIBRANT, ViralVerify, Virsorter2, and IPEV) showed BEREN outperforms these tools on both NCLDV contig and Preplasmiviricota genome retrieval from the mock metagenome. Out of the 637 NCLDV contigs above 10 kbp in the mock metagenome, BEREN was able to recover 489 contigs (77%) (Fig. 1b). The next best tool, geNomad, was able to identify 63% of these contigs, with many tools recovering below 50%. It is important to note that these contigs do not represent full NCLDV genomes, as many genomes contain multiple contigs.

Recovery of Preplasmiviricota genomes gave similar results as BEREN recovered all (100%) of the 100 genomes seeded into the mock metagenome (Fig. 1c). Other tools recovered between 4% and 83% of the Preplasmiviricota genomes.

### 3.2 BEREN recovers high-quality NCLDV genomes

Out of the 69 seeded NCLDV genomes, BEREN was able to recover 66 (95.6%). Using BLASTn to match the recovered genomes with the seeded genomes, we were able to calculate the completeness of each recovered genome. Genomes recovered with BEREN had an average completeness of 84% and a median completeness of 89% (Fig. 1d).

### 3.3 Testing BEREN with real-world metagenomes

As a demonstration of its potential use, BEREN was run on a metagenomic dataset from the Baltic Sea. The results from this analysis can be found in the supplementary text (Fig. S2).

## 4 Discussion

In addition to outperforming other viral identification tools in the realm of NCLDVs and Preplasmiviricota recovery, BEREN has several other advantages. One of such advantages is that BEREN is designed specifically for these two groups of viruses and, therefore, provides many helpful downstream outputs for studying their diversity, such as marker genes for phylogenetic and taxonomic assignment. While several other tools were able to recover NCLDVs and Preplasmiviricota viruses, many of them do not provide any distinguishing taxonomic or phylogenetic information about these viruses. For example, DeepMicroClass and IPEV label both these phyla as “Eukaryote Virus,” and other tools like VIBRANT and DeepVirFinder do not distinguish them from bacterial viruses.

Another advantage of BEREN is the ability to recover NCLDV genomic bins in addition to contigs. As far as we know, no other virus-identification tool tested can recover NCLDV genomic bins, which is a key limitation, as NCLDV genomes are often too large to be represented by a single contig (Aylward *et al.* 2021). Getting genomic bins also has the advantage of providing genome annotations to uncover the functional potential of these viruses.

## Supplementary Material

vbaf284_Supplementary_Data

## Data Availability

BEREN is an open-source tool available on Gitlab (https://gitlab.com/benminch1/BEREN/-/tree/master). All scripts to run the program, as well as test metagenomes, are available there. Genomes used in the mock metagenome, all alignments for custom HMM profiles, and phylogenetic trees can be found on figshare (https://figshare.com/projects/BEREN/249416).
