# Supplementary material for: BEREN: a bioinformatic tool for recovering giant viruses, polinton-like viruses, and virophages in metagenomic data
Source: Bioinform Adv. 2025 Nov 8;5(1):vbaf284. doi: 10.1093/bioadv/vbaf284 (PMC12638062; doi:10.1093/bioadv/vbaf284)
Supplement: vbaf284_Supplementary_Data [file vbaf284_supplementary_data.docx]

**Supplemental Text**

**Recovery of Mirusvirus and Mryiavirus Genomes and Markers**

The NCLDV bins module also has the possibility of recovering some Mirusviricota and the majority of the Mriyavirus genomes in a metagenomic dataset, as these viruses share many of the same marker genes and orthologous groups with their NCLDV counterparts (Gaïa *et al.*, 2023; Yutin *et al.*, 2024). Through testing, these bins typically end up being classified as partial genomes, as they have lower ViralRecall scores compared to NCLDVs and fewer identified marker genes (Figure S1a). A simple test using a mock metagenome assembly containing 111 Mirusvirus and 60 Mriyavirus genomes demonstrated BEREN was able to recover 31% of Mirusvirus genomes and 91% of Mriyavirus genomes (Figure S1b). These bins are flagged by BEREN and put into separate folders for further analysis at the discretion of the researcher.

The NCLDV marker module can also search for marker genes found in the newly discovered Egovirales order, proposed *Mriyaviricetes* class (Yutin *et al.*, 2024), and *Mirusviricota* phylum. The dataset is searched for *Mirusviricota* and Egovirales MCPs using hmm profiles obtained from Gaïa et al. (Gaïa *et al.*, 2023; Gaïa *et al.*, 2024) and *Mryiyaviricetes* MCPs using a custom hmm profile. All of these HMMs are leveraged in a custom script for parsing.

**BEREN comprehensively assesses the diversity and metabolic potential of eukaryotic viruses in a real-world metagenome.**

A publicly available metagenomic dataset from the Baltic Sea was downloaded to demonstrate a real-world application of the tool. This dataset consisted of ten metagenomes, five from the cellular fraction (>0.2uM) and five from the viral fraction (<0.2uM). BEREN was run with all NCLDV modules on the cellular fraction and the *Preplasmiviricota* module on the viral fraction.

A total of 482 quality-filtered NCLDV PolB marker genes were recovered from the Baltic datasets (Figure S2a). These represented all major NCLDV orders, with the majority being either Algavirales or Imitervirales. From the five cellular fraction samples, 492 NCLDV contigs were recovered, ranging from 10-117 kbp (Figure S2b). Many of these contigs contained multiple marker genes, with certain contigs containing up to 8. For contigs over 30kbp, the mean number of marker genes ranged from 1 to 4. 53 NCLDV bins were also recovered from the 5 cellular metagenomes, including 2 Pandoravirales bins, 21 Imitervirales bins, and 30 Algavirales bins (Figure S2c). These genomic bins ranged in size from 52-485 kbp. Applying the metabolism and protein annotation module on these genomes revealed a variety of metabolic genes such as those involved in light harvesting, DNA processing, and carbon metabolism (Figure S2c).

After running BEREN on the five viral-fraction samples, a total of 9 viruses of the phylum *Preplasmiviricota* were recovered (Figure S2d). These viruses ranged in size from 10.5-19.9 kbp and all encoded a major capsid protein as well as another marker gene. Using BEREN, two of these genomes were identified as Virophages, while the rest were most likely Polinton-like viruses (PLVs). Using a refined phylogenetic approach with reference Virophage and PLV sequences, we were able to confirm the results from BEREN as two of the MCPs clustered with Virophage reference sequences and the rest clustered with PLV references (Figure S3).

**A note on False positives and negatives**

To access the distribution of false positives and negatives given contigs of various lengths, a test dataset was generated with 1000 randomly cut-up contigs coming from reference giant virus genomes (for false negative identification) and 1000 randomly cut-up contigs coming from bacteria and phage genomes downloaded from NCBI (for false positive identification). These cut-up contigs ranged from 10,000 bp to 1 Mb, and an even distribution of sizes was used for analysis. These contigs were run through various tools, including BEREN in NCLDV contig mode, to assess the percentage of false positives and negatives. Overall, BEREN had a low false negative rate, with the only false negatives being small contigs < 20kbp (Figure S4a). This false negative rate was consistent with that of other tested tools. BEREN, along with most other tools, did not identify any false positives (Figure S4b). The user should be advised that smaller contigs may be missed when using BEREN due to the lack of NCLDV marker genes or identifiable features, as BEREN is a relatively conservative tool.

**Supplemental Methods**

*NCLDV Marker module*

The NCLDV marker module stands apart from other modules as it can provide a standalone diversity of all NCLDV in a given metagenome. Markers generated from this analysis can be further used to build phylogenetic trees to gain insight into the taxonomy of recovered NCLDVs. In the BEREN tool, a sample PolB tree is built, as this is a common marker used for assessing the diversity of NCLDVs in a metagenome (Monier et al., 2008; Kijima et al., 2021), but this methodology could be followed for other markers by the user.

The choice of markers to identify for this section was chosen based on previous research showing these 9 markers to be useful for phylogenetic analysis, as they are present in > 70% of NCLDV genomes and form separate clades from other domains of life and other types of viruses (Aylward et al., 2021).

*NCLDV Contig module*

The contig module allows users to go beyond simply categorizing the diversity of NCLDVs in their metagenome and begin to characterize the functional potential and genomic repertoires of these viruses. Contigs from this module typically represent pieces of NCLDV genomes, but they could represent full genomes if the input metagenome was generated using long-read sequencing technology. These contigs can be integrated into the metabolism module to look at the diverse functional potentials of NCLDVs in a desired metagenome.

*Preplasmiviricota module*

While we acknowledge that the Preplasmiviricota phylum contains other types of viruses (Adenoviruses & Tectiviruses), we have decided to focus BEREN on only the recovery of Polintons, PLVs, and Virophages, as these are widespread in many ecosystems and of ecological significance given their interactions with NCLDVs (Bellas & Sommaruga, 2021). Also, Tectiviruses infect bacteria, which is not the focus of the BEREN tool.


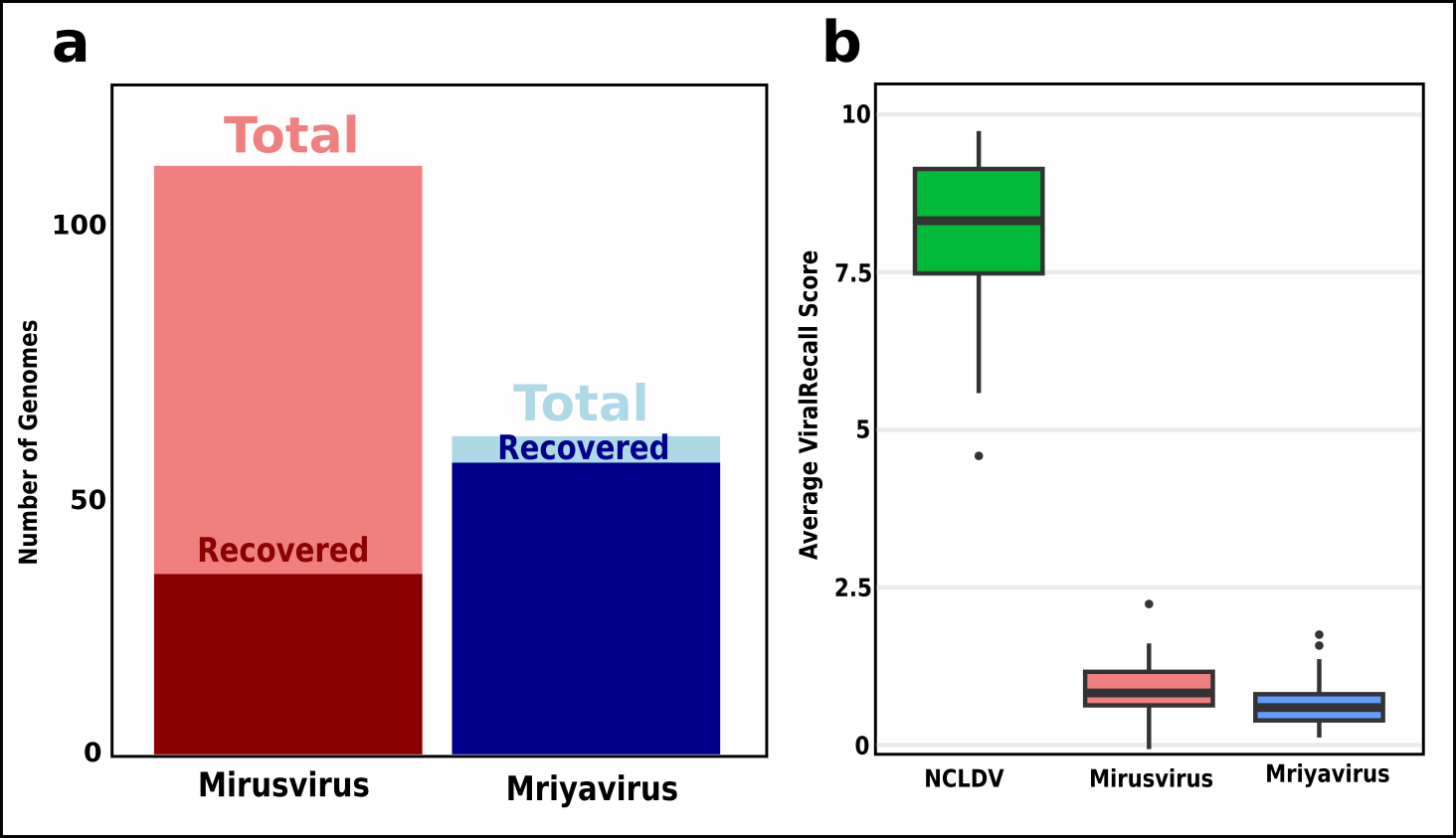


**Figure S1. BEREN’s ability to recover Mirusvirus and Mriyavirus genomes. (a)** A total of 111 Mirusvirus and 60 Mriyavirus genomes were used as input to BEREN as part of a mock metagenome. The total number of genomes recovered and identified by BEREN is shown above. **(b)** The average ViralRecall score for Mirusviruses and Mriyaviruses as compared to NCLDV genomes recovered using the BEREN pipeline.


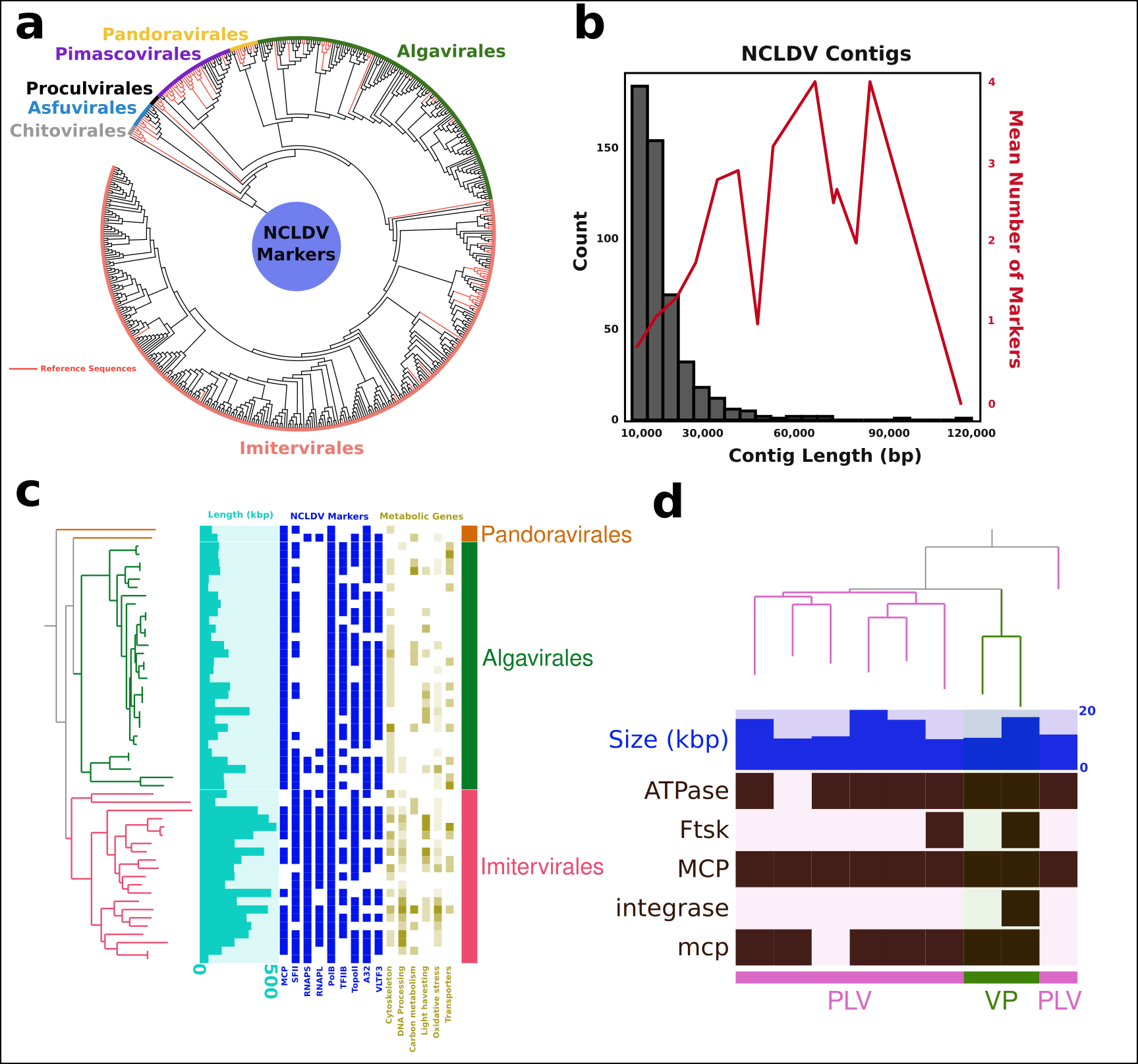


**Figure S2. Testing BEREN on real-world Baltic Sea metagenomes.** BEREN was used to assess the diversity of NCLDVs and *Preplasmiviricota* in 10 Baltic Sea metagenomes. **(a)** A phylogenetic tree of recovered NCLDV PolB marker genes, clustered with custom reference sequences from the GOEV database (ref). **(b)** NCLDV contigs recovered from the five cellular fraction metagenomes. The red line represents the mean number of NCLDV markers in a given “bin” or size class. **(c)** A phylogeny of recovered NCLDV genomes was created using the PolB marker gene. Genome length, NCLDV markers, and metabolic gene categories are also represented on this tree. **(d)** *Preplasmiviricota* genomes recovered from the viral fraction metagenomes were phylogenetically placed based on the major capsid protein (MCP). Other marker genes, as well as genome size, are present in the tree. *Preplasmiviricota* genomes are classified as Polinton-like virus (PLV) or Virophage (VP) by BEREN utilizing the virophage_affiliation script. An interactive version with tree labels of the NCLDV PolB tree is available on iTol (<https://itol.embl.de/export/1927017135431021751302943>).


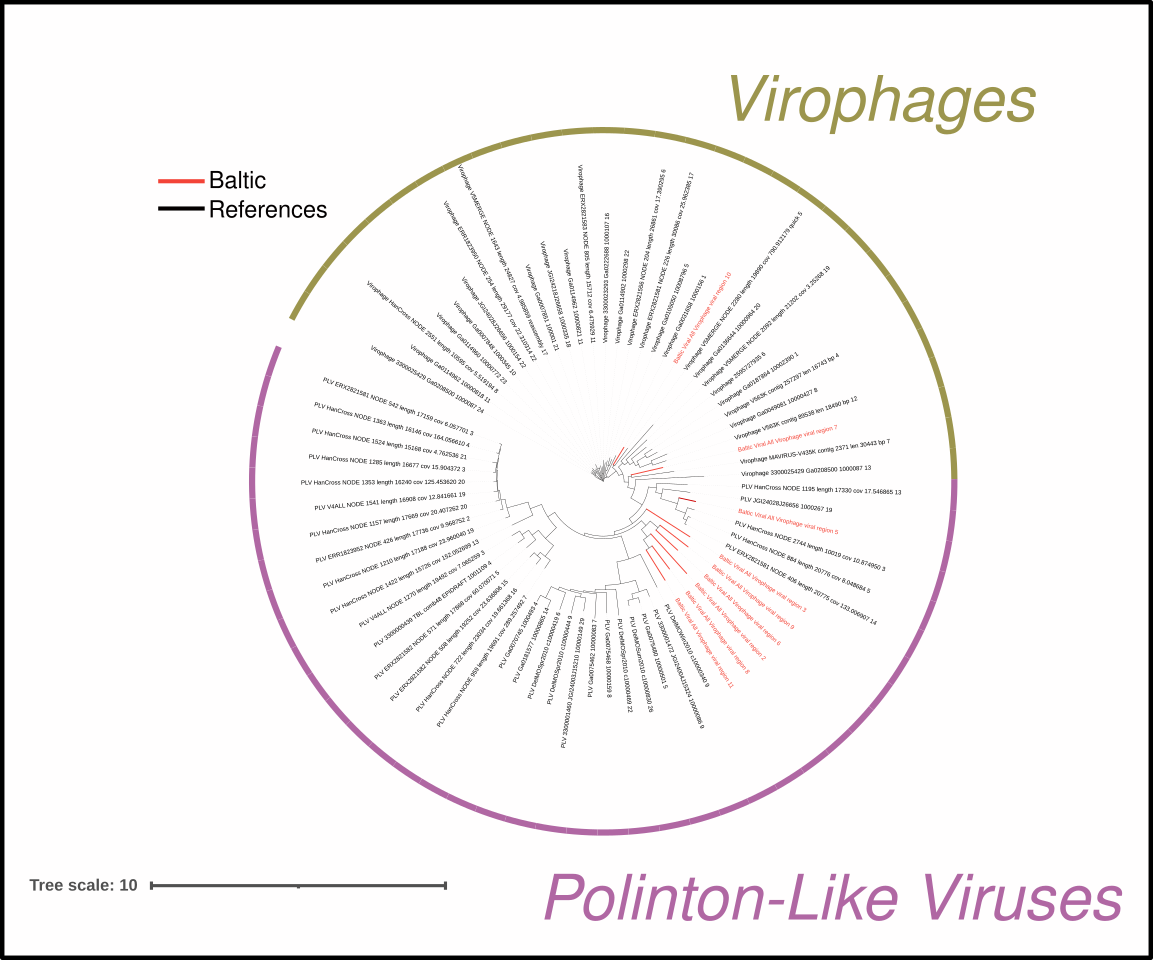


**Figure S3. Phylogenetic tree of reference PLVs/Virophages and genomes identified in this study.** A tree of virophage and Polinton-like virus (PLV) major capsid proteins (MCPs) recovered from the Baltic sea metagenomes. Reference sequences for Virophages and PLVs were retrieved from Bellas and Sommaruga (2021), and final trees were made through MAFFT for alignment and FastTree for tree-building. The tree was visualized in iTol, and an interactive version of the tree is available in this link: <https://itol.embl.de/export/1927017135247941751306766>


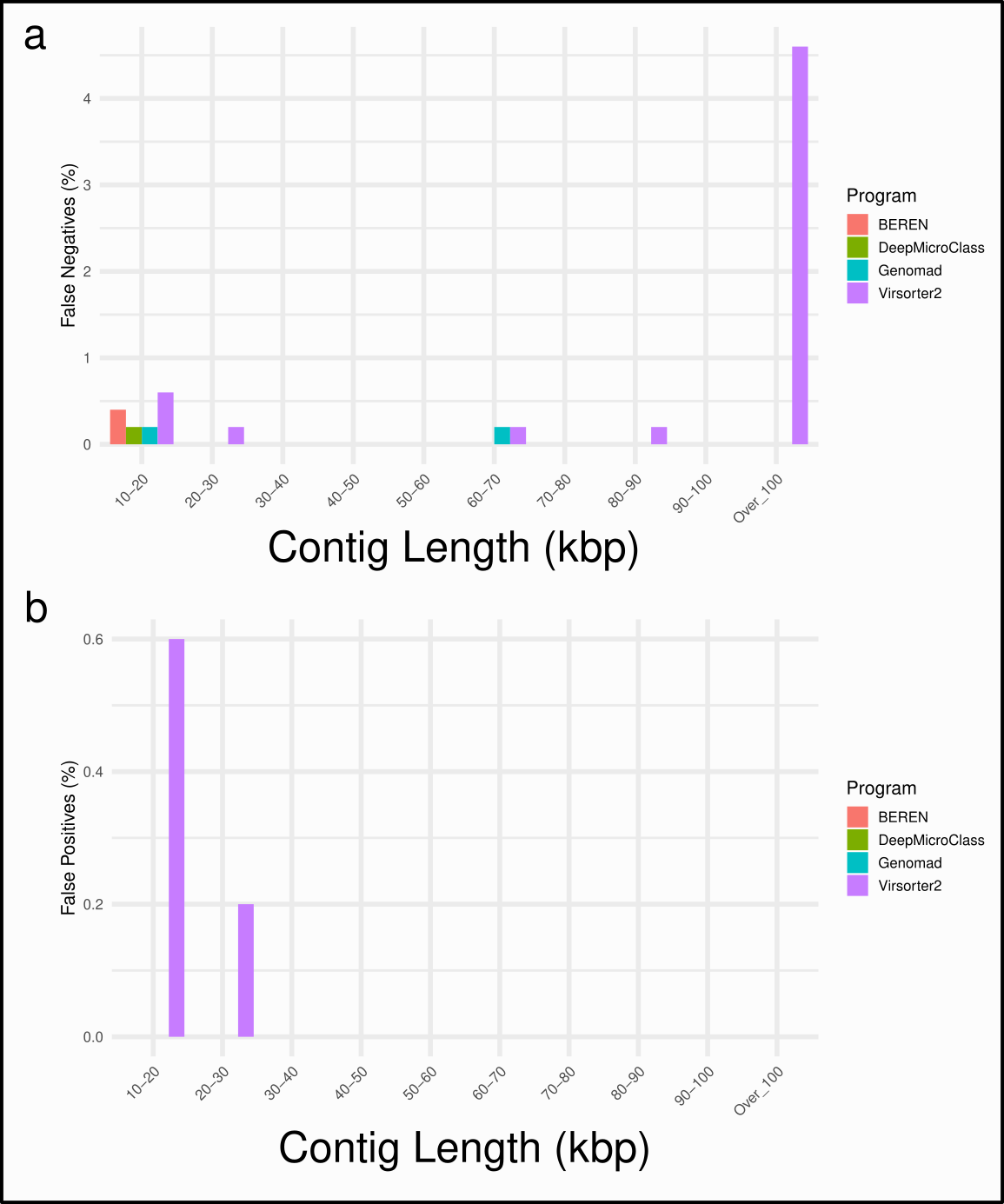
**Figure S4. An analysis of the false positive and false negative percentages of BEREN across contig lengths.** (a) False negative and (b) false positive rates were determined through the use of a mock dataset with known positives (giant viruses) and negatives (bacteria and bacteriophages). This dataset was cut up into various contig lengths from 10,000 bp to 1 Mbp and run through BEREN and other virus identification tools.

**References**

Aylward FO, Moniruzzaman M. ViralRecall—A flexible command-line tool for the detection of giant virus signatures in ‘omic data. Viruses. 2021;13(2):150. doi:10.3390/v13020150

Bellas CM, Sommaruga R. Polinton-like viruses are abundant in aquatic ecosystems. Microbiome. 2021;9:13. doi:10.1186/s40168-020-00956-0

Gaïa M, Ruscheweyh HJ, Eren AM, Koonin EV, Sunagawa S, Krupovic M, Delmont TO. Egoviruses: distant relatives of poxviruses abundant in the gut microbiome of humans and animals worldwide. bioRxiv. 2024 :2024-03.doi:10.1101/2024.03.23.586382

Gaïa M, Meng L, Pelletier E, Forterre P, Vanni C, Fernandez-Guerra A, Jaillon O, Wincker P, Ogata H, Krupovic M, Delmont TO. Mirusviruses link herpesviruses to giant viruses. Nature. 2023;616(7958):783-9.. doi:10.1038/s41586-023-05962-4

Kijima S, Delmont TO, Miyazaki U, Gaia M, Endo H, Ogata H. Discovery of viral myosin genes with complex evolutionary history within plankton. Frontiers in Microbiology. 2021 ;12:683294.

Monier A, Larsen JB, Sandaa RA, Bratbak G, Claverie JM, Ogata H. Marine mimivirus relatives are probably large algal viruses. Virology Journal. 2008 ;5(1):12.

Yutin N, Mutz P, Krupovic M, Koonin EV. Mriyaviruses: small relatives of giant viruses. mBio. 2024;15(7):e01035-24. doi:10.1128/mbio.01035-24
